# A Simple Method to Extract DNA from Hair Shafts Using Enzymatic Laundry Powder

**DOI:** 10.1371/journal.pone.0069588

**Published:** 2013-07-29

**Authors:** Zheng Guan, Yu Zhou, Jinchuan Liu, Xiaoling Jiang, Sicong Li, Shuming Yang, Ailiang Chen

**Affiliations:** Institute of Quality Standards and Testing Technology for Agro-products, Key Laboratory of Agro-product Quality and Safety, Chinese Academy of Agricultural Sciences, Beijing, China; Key Laboratory of Agro-food Quality and Safety, Ministry of Agriculture, Beijing, China; Natural History Museum of Denmark, Denmark

## Abstract

A simple method to extract DNA from hair shafts was developed by using enzymatic laundry powder at the first step of the process. The whole extraction can be finished in less than 2 hours. The simple extraction reagent proposed here contains only two cheap components: ordinary enzymatic laundry powder and PCR buffer. After extraction, an ultra sensitive fluorescent nucleic acid stain, PicoGreen, was used for quantifying trace amount of double-stranded DNA in the solution extracted. For further validation of DNA extraction, four primers were employed to amplify DNA microsatellite loci. Both fluorescence spectroscopy and PCR results suggested that this method can extract DNA from hair shafts with good efficiency and repeatability. The study will greatly facilitate the use of hair shafts in future for DNA analyses on genome-wide scale.

## Introduction

Hair shafts as a potential source of DNA is valuable for noninvasive study of human and nonhuman populations. It can be used in genetic analysis to identify individuals and breed in animal husbandry traceability, wild animal germplasm resources protection, forensic medical study and paleontological research, etc. The characteristic of mitochondrial DNA (mtDNA) extracted from hair shafts has already been well described previously [1]–[5]. In addition, James Robertson *et al.* [6] described that mtDNA has limited value because mtDNA profiles cannot be compared with national and international databases of Short Tandem Repeat (STR) genotypes. Furthermore, mtDNA cannot discriminate maternal relatives and lack the discriminating power of STR profiles. However, fragmentation of the nuclear DNA (nuDNA) in the hair shafts was considered as a result of the keratinisation process [7] and nuDNA seldom been successfully analyzed. Thus, a major concern in the hair shafts DNA extraction and amplification today is trying to find an efficient, stable and easy way to extract DNA and amplify nuDNA targets.

In the present study, we have developed a simple method to extract DNA from hair shafts. With this approach, it took less than 2 hours to extract DNA and the simple extraction reagent contains only ordinary enzymatic laundry powder and 1× PCR buffer, which was cheap and easily available. Fluorescence spectroscopy approach, Polymerase Chain Reaction (PCR) and on-chip-electrophoresis results confirmed that enzymatic laundry powders could be used to extract DNA and amplify microsatellite markers from hair shafts.

## Materials and Methods

### Ethics Statement

The study was approved by the Animal Care and Use Committee of the Institute of Quality Standards and Testing Technology for Agro-products, Chinese Academy of Agricultural Sciences and the owners of the cattle. Permission to collect and use hair shafts samples from cattle in this study were granted by Shandong Kelong Farming Industry Limited Company.

### Samples

Hair shafts were collected from cattle (24 pure bred Luxi cattle, in Heze, Shandong Province, China), November 2011. Fresh liver samples of beef cattle were purchased from Beijing Anding Abattoir. The samples were stored at −18°C until DNA extraction.

### DNA extraction

Full precautions were taken to prevent external DNA contamination. All experiments were performed following protocols for ancient DNA extraction[8], [9]. Laboratories, laboratory ware, plastic ware, reagents, pipettes, pipette tips, benches and equipments were irradiated under ultraviolet light (250 nm for 1 hour) before and after each experiment. In addition, only the ware and pipette tips, which have been steam sterilized (high steam pressure, 121°C for 1 hour) were used in this study. All the steps of the analysis were performed wearing sterile gloves, face masks, hair nets and laboratory coats, under positive pressure in a laminar flow hood. Reagents are molecular grade except enzymatic laundry powder. Negative controls (containing a reagent blank) were included in all assays. Prior to DNA extraction, samples were decontaminated through immersion in 84™ Disinfectant (the content of effective chlorine is 5–6%) for 30–40 seconds, then rinsed in ddH_2_O three times. The ddH_2_O, which was used to rinse the sample (the third time), was set as a pre-treatment control to ensure there was no contamination. After that, samples were soaked in sterile ethanol, and left to air-dry.

Hair shafts were cut into fragments of about 2 mm. Each sample (Table 1 and Table 2) was digested in 100 µl of extraction reagent (pH 10.3) for 1.5 hours at 50°C. The extraction reagent contained 3 mg enzymatic laundry powder (Diao™ enzymatic laundry powder made by Nice Group, Keon™ enzymatic laundry powder made by Nafine, and OMO™ enzymatic laundry powder made by Unilever), and 1×PCR buffer (20 mM Tris-HCl (pH 8.4), 20 mM KCl, 10 mM (NH_4_)_2_SO_4_, 1.5 mM MgCl_2_, Tiangen Biotech (Beijing) Co., Ltd.). Besides that, liver DNA was separately extracted in another room using Genomic DNA Purification Kit (Fermentas Life Sciences, Thermo Fisher) as a positive control.

**Table 1:** Experimental design of DNA extraction for PCR and on-chip-electrophoresis.

Microsatellite marker*	*CSRM60* and *INRA035*
Cattle Quantity	12 cattle each marker
Sample Weight	1 mg
Extraction Solution	Diao™ enzymatic laundry powder
Extraction Volume	100 µl
PCR Template Amount (1st round)	0.1 µl
PCR Template Amount (2nd round)	2 µl

* ISAG-FAO recommended microsatellite markers for cattle.

**Table 2:** Experimental design of DNA extraction for real-time PCR

SampleWeight (mg)	Volume (µl)	Extraction Reagent Groups*	PCR Template Amount Groups (µl)		Microsatellite marker*	Cattle Quantity
			1st round	2nd round		
5	100	D, K and O	5, 2, 1, 0.5, 0.2, 0.1	2	*ETH225* and *HAUT27*	24
2	100	D, K and O	5, 2, 1, 0.5, 0.2, 0.1	2	*ETH225* and *HAUT27*	24
1	100	D, K and O	5, 2, 1, 0.5, 0.2, 0.1	2	*ETH225* and *HAUT27*	24
0.5	100	D, K and O	5, 2, 1, 0.5, 0.2, 0.1	2	*ETH225* and *HAUT27*	24
0.2	100	D, K and O	5, 2, 1, 0.5, 0.2, 0.1	2	*ETH225* and *HAUT27*	24
0.1	100	D, K and O	5, 2, 1, 0.5, 0.2, 0.1	2	*ETH225* and *HAUT27*	24

* ISAG-FAO recommended microsatellite markers for cattle. D: Diao™ enzymatic laundry powder. K: Keon™ enzymatic laundry powder. O: OMO™ enzymatic laundry powder. Each sample weight group has 3 extraction reagent groups (8 cattle for each extraction reagent group). For PCR, each sample was set 6 PCR template amount groups in 1^st^ round.

After extraction, extraction solutions were gradually heated up to 95°C to improve extract efficiency[10], and then subject to 95°C for 10 minutes in order to inactivate enzymes in the extraction reagent. The final DNA extracts were stored at −18°C until use.

### DNA quantitation

Quant-iT™ PicoGreen® dsDNA reagent (Invitrogen) is an ultra-sensitive fluorescent nucleic acid stain for quantitating double-stranded DNA (dsDNA) in solution. It is used to help quantify DNA in extraction solution by fluorescence spectroscopy approach. This method is at least 400 times more sensitive than the Hoechst dye based assay [11].

The quantification assay was carried out as described in Chinese Pharmacopoeia (Appendix B, Vol. III)[12] which states that the lower limit of detection of this approach is 0.3 ng/ml and linearity is reported between 1.25–80 ng/ml. Therefore according to the results of published literature[6], [13], [14], the DNA in hair shafts could be detected by the quantitative assay.

### Microsatellite amplification

PCR reactions were carried out in 20 µl reaction volumes. Each PCR reaction contained 0.5 unit HotStar Taq® DNA Polymerase (QIAGEN), 200 µM dNTP Mixture (Tiangen Biotech (Beijing) Co., Ltd.), 1×PCR buffer (Tiangen Biotech (Beijing) Co., Ltd.), 200 nM each primer, and different volumes of template (Table 1). For real-time PCR, it was carried out in 20 µl reaction volumes with the final concentration of 1× Power SYBR Green PCR Master Mix (ABI), 200 nM each primer, and different volumes of template (Table 2). A joint committee of Food and Agriculture Organization of the United Nations (FAO) and the International Society for Animal Genetics (ISAG) has recommended a list of microsatellite markers for cattle[15]. For the amplification, four markers (*CSRM60*, 79–115 base pairs (bp); *INRA035*, 100–124 bp; *ETH225*, 131–159 bp; and *HAUT27*, 120–158 bp) were selected from the list and the primers were synthesized by Shanghai SANGON Biological Engineering Technology Services Limited.

The extract solution was used directly as PCR template in the first round. The second round used the product of the first round as its template. The two-round cycling steps were identical and as follows: initial denaturation at 95°C for 10 minutes, followed by 35 cycles at 95°C for 20 seconds, annealing (40 seconds, annealing temperatures are shown in Table 3), extension at 72°C for 1 minute, and a final extension at 72°C for 10 minutes.

**Table 3:** Annealing temperatures.

Microsatellite marker*	Annealing Temperature
*ETH225*	62°C
*CSRM60*	
*HAUT27*	57°C
*INRA035*	

* ISAG-FAO recommended microsatellite markers for cattle.

Positive control was amplified in another room, with same condition and same template volume but only the first round PCR was performed.

Then, amplification products (locus *CSRM60* and *INRA035*) were pipette onto DNA Chips (on-chip-electrophoresis, Agilent DNA 1000 Kit, for use with the Agilent 2100 bioanalyzer) and operated as its Guide described, while the others were visualized under UV light on 1.2% agarose gels by staining with GeneGreen (Tiangen Biotech (Beijing) Co., Ltd.).

### Statistical Analysis

All statistical analyses were performed with IBM SPSS Statistics (Version 19) software. Calculations were done in Microsoft EXCEL spread sheets. The arithmetic means and standard deviations were used to carry out a standard t-test, and the significance level was measured at less than 0.05. Moreover, Spearman's rho test is used for detecting correlation between the C_T_ values and input DNA amounts.

## Results

**Figure 1:**
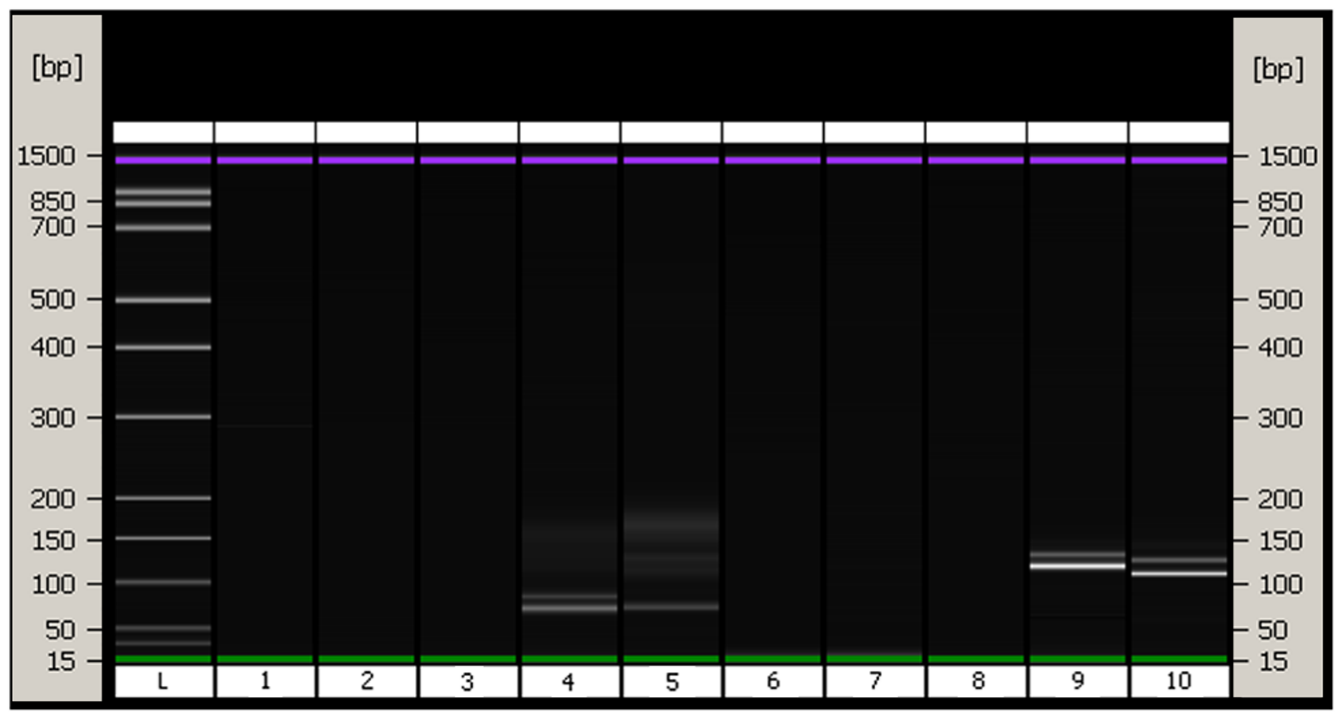
Amplification results using the proposed DNA extraction method (on-chip- electrophoresis results). The size of PCR products are 79–115 bp (*CSRM60*), 100–124 bp (*INRA035*). Left to right: L, ladder; 1, sample washing water control (*CSRM60*); 2, extraction solution (without hair shaft) control (*CSRM60*); 3, ddH_2_O control (*CSRM60*); 4, DNA from hair shafts of pure bred Luxi cattle (*CSRM60*); 5, DNA from liver of beef cattle (*CSRM60*); 6, sample washing water control (*INRA035*); 7, extraction solution (without hair shaft) control (*INRA035*); 8, ddH_2_O control (*INRA035*); 9, DNA from hair shafts of pure bred Luxi cattle (*INRA035*); 10, DNA from liver of beef cattle (*INRA035*).

**Figure 2:**
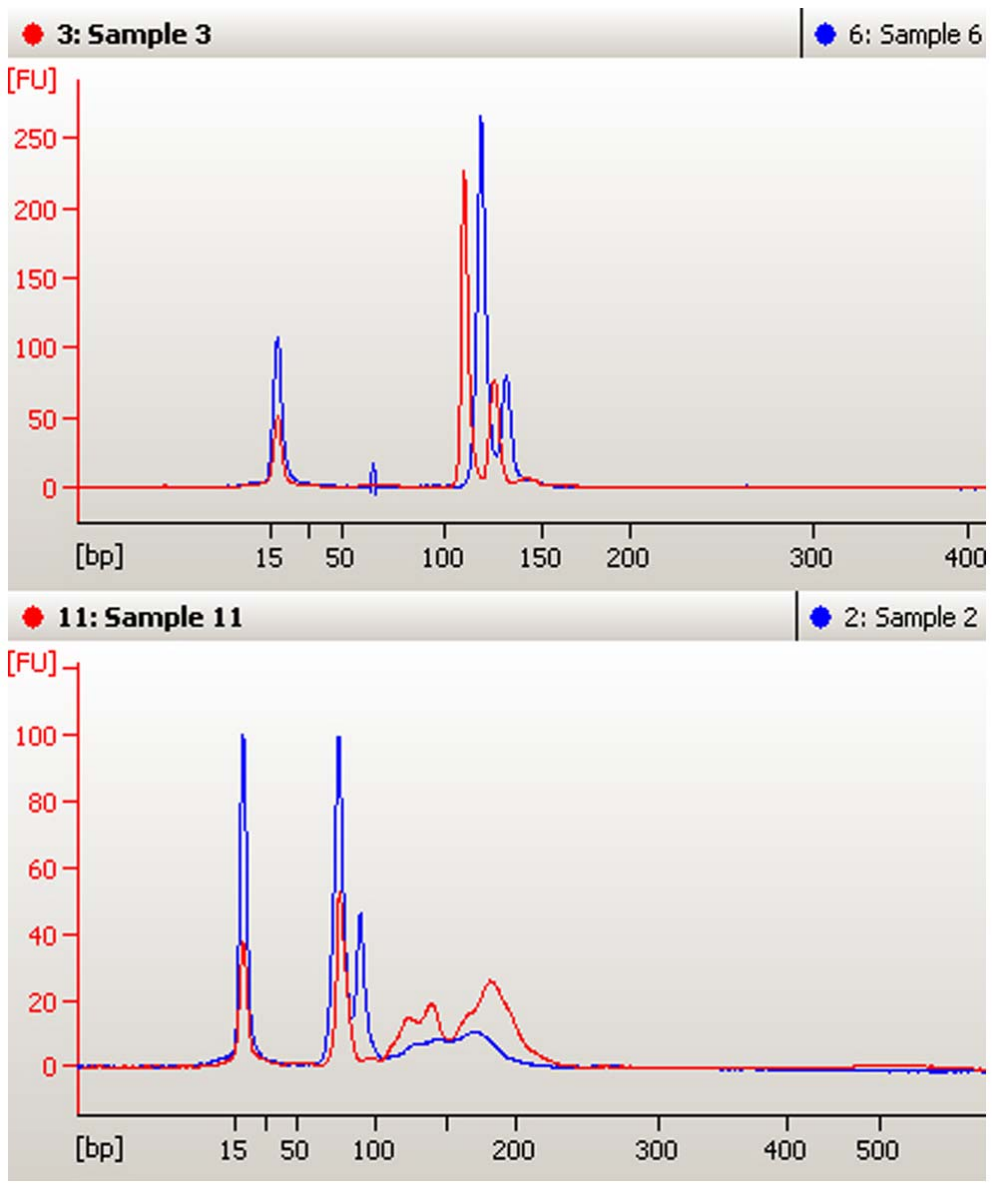
Comparison of amplification results of DNA extracted from hair shafts using the proposed method and that extracted from liver using commercial Genomic DNA Purification Kit (on-chip-electrophoresis results). The above panel is *INRA035* comparison result, sample 6 is amplification result of hair shaft DNA from pure bred Luxi cattle and sample 3 is amplification result of liver DNA from beef cattle; The below panel is *CSRM60* comparison result, sample 2 is amplification result of hair shaft DNA from pure bred Luxi cattle and sample 11 is amplification result of liver DNA from beef cattle.

DNA was extracted from hair shafts of 24 pure bred Luxi cattle. The DNA content of the extract (Table 1) is 8.87±0.50 ng/ml (n = 24) and the values of negative control groups are below the limit of detection. The samples were randomly divided into two groups to amplify locus *CSRM60* and *INRA035* respectively. 11 of the 12 samples were amplified *CSRM60* successfully, and 9 of the other 12 samples were amplified *INRA035* successfully. No blanks exhibited evidence of contamination. Reagent blank (extraction solution without hair shafts), negative control (ddH_2_O as template), and pre-treatment control (the third time washing water of hair shafts after disinfectant treatment) confirmed the absence of any external DNA contamination by the results of fluorescence spectroscopy and on-chip-electrophoresis identification tests. On-chip-electrophoresis results were shown in Figure 1. The bands of chip-electrophoresis of hair shaft DNA amplification results were compared with that of liver source DNA in Figure 2 and the results demonstrated the accuracy of our target locus according to the ISAG-FAO document[15].

**Figure 3:**
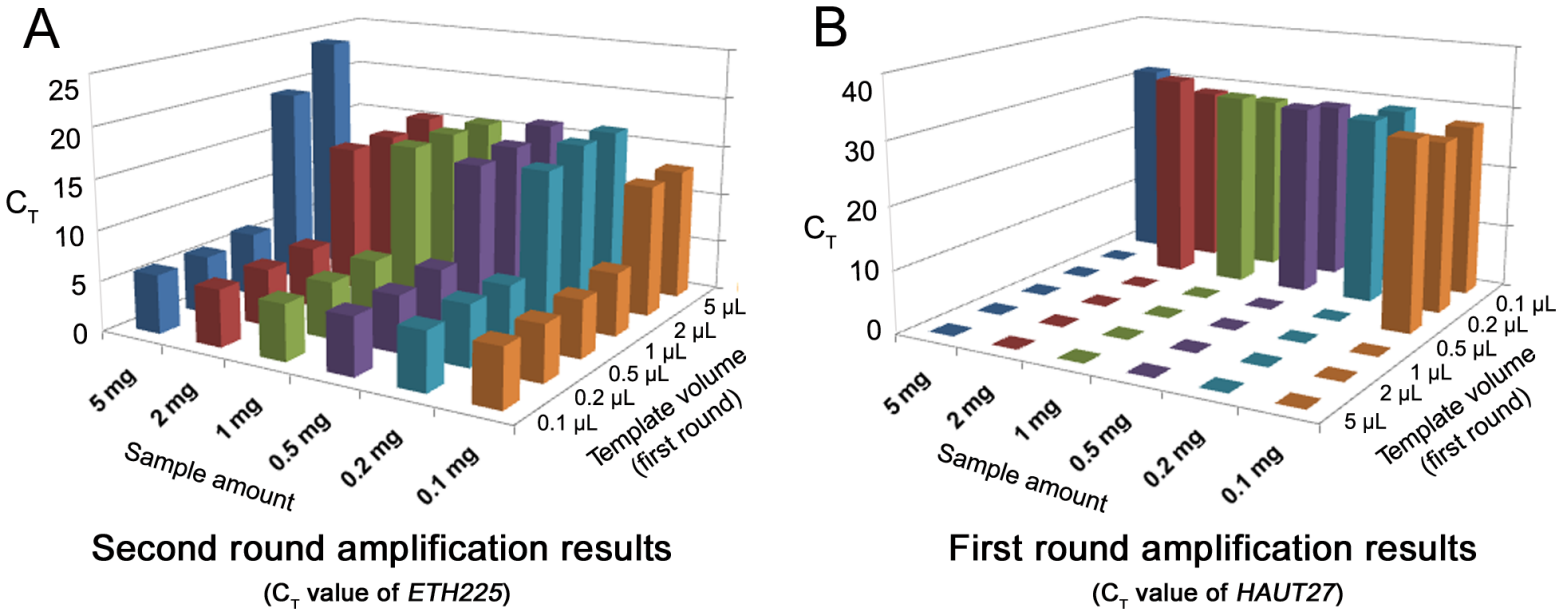
Effects on the efficiency of real-time PCR of sample amount used for extraction and input template volume. (A) Second round amplification result (C_T_ value of *ETH225*). Six sample amount groups (5 mg, 2 mg, 1 mg, 0.5 mg, 0.2 mg, 0.1 mg) and six template volume groups (different template volumes in first round, but same template volume (2 µl) in second round) of Diao™ enzymatic laundry powder. (B) First round amplification result (C_T_ value of *HAUT27*). Six sample amount groups (5 mg, 2 mg, 1 mg, 0.5 mg, 0.2 mg, 0.1 mg) and six template volume groups of Diao™ enzymatic laundry powder. Because of amplification inhibition factors, no C_T_ value of 5 µl, 2 µl, 1 µl template group and some of 0.5 µl and 0.2 µl template groups were obtained (“undetermined”). Negative controls are not shown in this figure; their C_T_ values were undetermined too.

**Figure 4:**
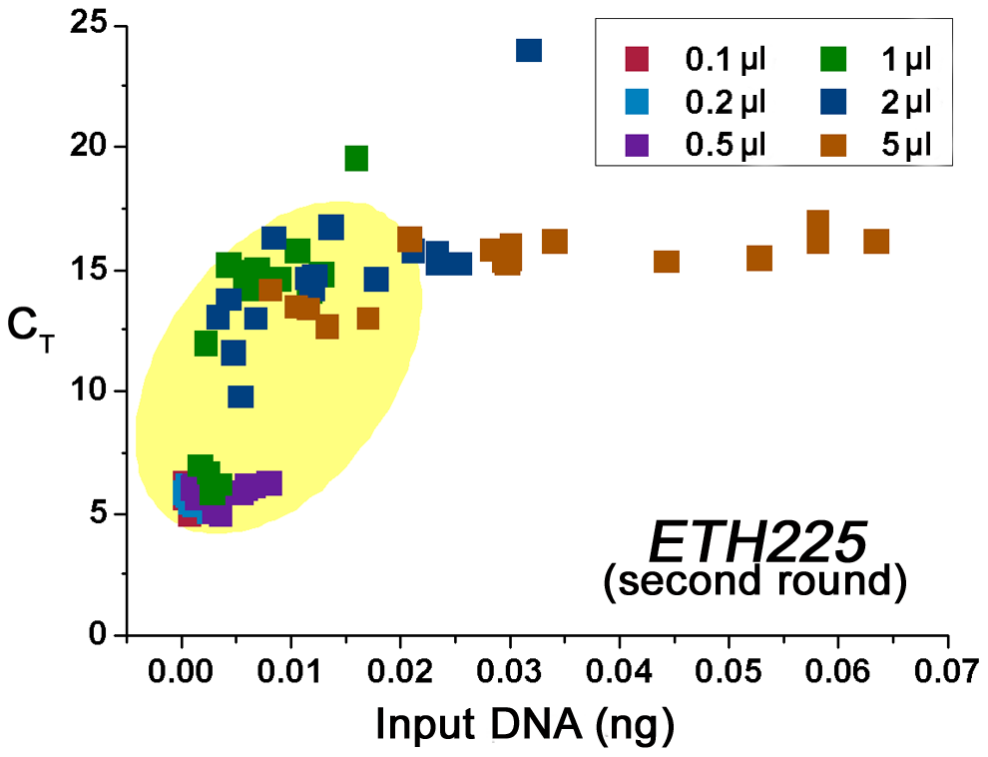
Negative correlation between PCR efficiency and the amount of input template. The data of all three enzymatic laundry powder groups (each detergent group has six sample amount groups: 5 mg, 2 mg, 1 mg, 0.5 mg, 0.2 mg, 0.1 mg; and each sample amount group has six template volume groups: 5 µl, 2 µl, 1 µl, 0.5 µl, 0.2 µl, 0.1 µl) were adopted (target locus *ETH225*, second round), Spearman's rho = 0.810, P<0.001, n = 107 (one sample has no C_T_ value and has been excluded by the SPSS). 90 of 107 points were in circular ROI box (yellow oval area), mean C_T_ value is 8.7, SD = 4.1; and median C_T_ value is 6.0.

Moreover, to investigate the best template volume and adequate sample amount, six template volume groups and six sample weight groups were set to amplify locus *ETH225* and *HAUT27* respectively. In this part, we extracted 144 samples (Table 2), and as shown in Table 4, all of them can yield DNA by using the proposed extraction method with enzymatic laundry powder. The results of real-time PCR (Figure 3, Figure 4 and Table 5) demonstrated that low template amount groups were amplified more efficiently. Aside from the template amount in the amplification, for this approach, sample amount is not a major factor.

**Table 4:** DNA yield of extraction with different enzymatic laundry powder and sample amounts.

Groups	Diao™ enzymatic laundry powder (ng/ml)	Keon™ enzymatic laundry powder (ng/ml)	OMO™ enzymatic laundry powder (ng/ml)
0.1 mg sample	3.38±2.55^b^	2.30±0.52	1.61±0.28
0.2 mg sample	4.14±0.68^a^ ^b^	2.65±0.50	2.08±0.95
0.5 mg sample	12.69±4.14^a^ ^b^	6.80±1.55	5.94±1.21
1 mg sample	10.56±3.34^a^ ^b^	5.87±2.14	6.00±1.13
2 mg sample	8.86±1.58	5.66±1.49	5.98±1.90
5 mg sample	15.82±4.60	11.61±2.64	11.62±2.36

a *P*<0.05, compared with Keon™ enzymatic laundry powder group;

b *P*<0.05, compared with OMO™ enzymatic laundry powder group (*X* ¯± SD, *n* = 8).

**Table 5:** Results after two rounds of Real-time PCR using different enzymatic laundry powder, sample amounts and template volume in the first round PCR.

		*ETH225*						*HAUT27*					
		Template volume in the 1st round (μl)											
Extraction Reagent*	Sample (mg)	0.1	0.2	0.5	1	2	5	0.1	0.2	0.5	1	2	5
D	Neg (0)	×	×	×	×	×	×	×	×	×	×	×	×
D	0.1	√	√	√	√	√	×	√	√	√	√	√	×
D	0.2	√	√	√	√	√	×	√	√	√	√	×	×
D	0.5	√	√	√	√	√	×	√	√	√	×	×	×
D	1	√	√	√	√	√	×	√	√	√	×	×	×
D	2	√	√	√	√	√	×	√	√	√	×	×	×
D	5	√	√	√	√	×	×	√	√	×	×	×	×
K	Neg (0)	×	×	×	×	×	×	×	×	×	×	×	×
K	0.1	√	√	√	√	√	×	×	×	√	√	√	×
K	0.2	√	√	√	√	√	×	√	√	√	√	√	×
K	0.5	√	√	√	√	√	×	√	√	√	√	×	×
K	1	√	√	√	√	√	×	√	√	√	×	×	×
K	2	√	√	√	√	√	×	√	√	√	×	×	×
K	5	√	√	√	√	√	×	√	√	√	×	×	×
O	Neg (0)	×	×	×	×	×	×	×	×	×	×	×	×
O	0.1	√	√	√	√	√	×	√	√	√	√	√	×
O	0.2	√	√	√	√	√	×	√	√	√	√	√	×
O	0.5	√	√	√	√	√	×	√	√	√	√	×	×
O	1	√	√	√	√	√	×	√	√	√	×	×	×
O	2	√	√	√	√	√	×	√	√	√	×	×	×
O	5	√	√	√	√	√	×	√	√	√	×	×	×

√ Amplified successfully; × Failed amplification. Neg, reagent blanks. D: Diao™ enzymatic laundry powder. K: Keon™ enzymatic laundry powder. O: OMO™ enzymatic laundry powder.

## Discussion

The fact that enzymatic laundry powder contains protease, lipase, cellulose, surfactant and suspending agent, suggests that it could be used as a good extraction reagent to extract DNA [16] from the hair shafts. In the study, all three brands of enzymatic laundry powder contain anionic surfactant (sodium dodecyl benzene sulfonate), nonionic surfactant (polyoxyethylene ethers), suspending agent, and complex enzyme (including protease), that endow them with the extraction capability. Compared to the other methods (Table 6), it was not only simple and time-efficient, but also showed to be the most cost-effective method.

**Table 6:** Comparison of different DNA extraction methods.

DNA extraction method	QIAamp DNA Micro Kit (QIAGEN) extraction	Organic extraction (traditional method)	Enzymatic laundry powder extraction
Time	∼1 hour	∼18 hours (including overnight incubation)	∼2 hours
Cost (100 samples)	¥4720.00	<¥1600.00	<¥70.00
Advantages	Rapid protocol; high recovery of DNA; safe; extractions were purified	Inexpensive; extractions were purified	Simple and rapid protocol; No DNA losing; safe; inexpensive
Disadvantages	More expensive than non-kit methods; possibly DNA losing	Many steps; overnight incubation; use of toxic chemicals; possibly DNA losing	Possibly inhibition during downstream molecular applications

**Figure 5:**
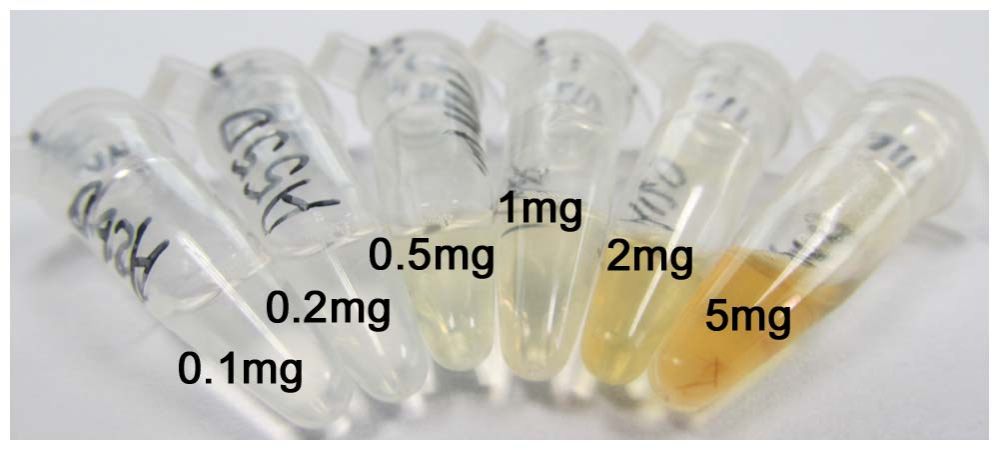
Photographic image of the extraction solution with different sample amounts.

NuDNA in hair shafts is known for its small quantity [17], and there are large quantities of impurities like keratin and pigments (Figure 5) which can affect the detection of nucleic acids and seriously inhibit PCR amplification. Ovoid pigment granules are located in the cortex (inner layer) and medulla (core), but the exact location of recoverable nuDNA in the hair shafts remains unclear. According to the literature [14], [18]–[20], we supposed that it is localized in the cuticle (outer layer). For this reason, we tried enzymatic laundry powder with the assumption that it can get enough nuDNA from cuticle as PCR template but less amplification inhibition substances.

As Nozawa and Uchihi *et al.* [18], [21] noted, the impurities like pigments can essentially give the same spectrum between 500 and 230 nm. Therefore, the spectrum of the DNA extract always appears to contain pigments in addition to DNA and some other substrates which have absorbance at wavelengths lower than 230 nm. It was challenging to quantify DNA amount in extraction solution directly before or without PCR except if the extract has been purified strictly. Another limitation was that sometimes the quantity of hair shafts or samples used for extracting DNA is not enough and the extracted DNA cannot reach the level of detectability (2 ng/µl) used by most detectors. Lack of DNA quantity in the extract leads to more failures in next PCR or other downstream work. Therefore, for the first time, we attempted fluorescence spectroscopy approach in this field and used Quant-iT™ PicoGreen® dsDNA reagent, an ultra-sensitive fluorescent nucleic acid stain to quantify dsDNA in extraction solution.

In this study, it has been proved that the sensitive fluorescent assay can be used to detect the DNA in hair shafts extraction solution directly, and enzymatic laundry powder can be used to extract DNA from the hair shafts. From the results obtained by fluorescent quantification, although the proposed method showed low DNA yield, the further PCR results suggested that it is enough to amplify target loci (*CSRM60*, *INRA035*, *ETH225*, and *HAUT27*). Furthermore, the following PCR with high success rate suggested that the low template amount could increase PCR efficiency (Figure 3, Figure 4 and Table 5). Actually, there is a negative correlation between PCR efficiency and input template amount and small amount of input DNA can decrease the C_T_ values (Figure 4, Spearman's rho = 0.810, P<0.001, n = 107). The results indicated that there were potential PCR inhibitors in the extraction solution which are the critical factors for successful PCR in our strategy. It is for that very reason that we can amplify targets successfully by using trace input DNA as low as 0.16 pg, and this also made it possible for most of enzymatic detergents to use in our method, although their extraction efficiency has significant differences (Table 4 and Table 5). According to the literature [18], [21]–[23], we considered that as a compromise between PCR template and amplification inhibition factors.

The extraction and two rounds amplification strategy presented above has been performed hundreds of times, but there still a few target loci failed to be amplified, we thought that it may be not caused by unpurified PCR template or non-optimized amplification condition, since it was clear that most of target genes can be amplified successfully. It might be caused by the keratinisation process for its cytolysis[24]–[26]. If the template was cracked, an amplification failure would occur as we described. Therefore, the proposed method using low amount DNA to overcome impurities inhibition on PCR may not of universal appeal e.g. in applications such as Next Generation Sequencing where maintaining overall molecular diversity is essential.

In conclusion, we developed a simple and cost-effective method to extract DNA from hair shafts using enzymatic laundry powder and PCR buffer with high efficiency and even can extract DNA from trace amount (0.1 mg) of hair shafts. The extract could be directly used as template to amplify the target gene. Repeated experiments also showed the good stability and reliability of the approach. The results indicated the simple method has a good practical future regarding its cost-effectiveness and easily operation. Considering hair shafts characteristics of easy to get, transport and store, we believe the proposed method in this study will find more applications in the animal husbandry traceability research, breeding cultivation and wild animal biodiversity study, etc.
